# One-Step Synthesis of Tea Polyphenol–Iron Nanoparticles for Enhanced Antioxidant and Antibacterial Properties

**DOI:** 10.3390/foods14244337

**Published:** 2025-12-16

**Authors:** Zhiwen Hu, Zhenzhen Wu, Lingxin Yu, Shuyi Zou, Yaxuan Hu, Tengjun Jiang, Yanlei Lin, Yueyue Cai, Qibiao Weng, Jie Pang, Jiebo Chen

**Affiliations:** 1 College of Food Science, Fujian Agriculture and Forestry University, Fuzhou 350002, China; 2 State Key Laboratory of Advanced Papermaking & Paper-Based Materials, College of Light Industry and Engineering, South China University of Technology, Guangzhou 510640, China; 3 College of Biological Science and Engineering, Fuzhou University, Fuzhou 350122, China; 4 Rural Revitalization College, Fujian Agriculture and Forestry University, Fuzhou 350002, China; 5 Fujian Provincial Key Laboratory of Eel Breeding and Processing, Fuzhou 350002, China; 6 National Engineering Research Center of Sugarcane, Fujian Agriculture and Forestry University, Fuzhou 350002, China; jiebo-chen@fafu.edu.cn

**Keywords:** green synthesis, tea polyphenol, iron, nanoparticles, characterization, antioxidant activity, antibacterial activity

## Abstract

Tea polyphenols (TPs) are promising natural bioactive compounds; however, their practical application is hindered by poor stability and low bioavailability. To address this challenge, we synthesized TP–iron nanoparticles (TP-Fe NPs) through coordination-driven self-assembly. Comprehensive characterization (SEM, TEM, FTIR, and XRD) confirmed the successful formation of stable TP-Fe NPs, primarily mediated by phenolic hydroxyl and carbonyl groups. Among TP-Fe NPs, the TP3-Fe1 NPs exhibited superior performance, achieving DPPH and ABTS radical scavenging rates of 65.71% and 89.64%, respectively, and inhibition rates of 91.44% against *E. coli* and 88.67% against *S. aureus*. Furthermore, TP3-Fe1 NPs demonstrated excellent biocompatibility, showing no significant cytotoxicity to L929 cells at 0.01–0.1 mg/mL. These findings highlight the potential of TP3-Fe1 NPs as a safe and effective material with dual functionality for antioxidant and antibacterial applications.

## 1. Introduction

Globally, consumers are increasingly concerned about food safety, quality, and waste reduction. This trend continues to drive technological innovations in food science and engineering. In this context, the development of novel, efficient, and safe antibacterial food materials has gained particular importance [1]. Such materials can not only extend food shelf life and preserve freshness but also effectively reduce food losses throughout production, storage, and distribution. As a result, research in this area has become a key frontier in food science and technology. Tea polyphenols (TP) have become very attractive and useful ingredients for food packaging, owing to their remarkable biological activities—including antioxidant, broad-spectrum antibacterial, and antiviral properties—coupled with their natural origin, safety, and renewability [2,3]. However, the practical application of TP is hampered by two major limitations: poor stability and low bioavailability. The phenolic hydroxyl groups in TP are susceptible to oxidation, polymerization, or degradation when exposed to light, heat, oxygen, or pH variations, leading to loss of bioactivity [4]. Furthermore, their limited solubility, dispersibility, and targeting ability within complex food matrices restrict effective interaction with microorganisms or oxidation sites, thereby diminishing their practical efficacy [5].

Nanoencapsulation has proven to be an effective strategy in terms of addressing these challenges. This approach involves loading TP into nanocarriers (e.g., nanoparticles, liposomes, nanoemulsions) or leveraging TP’s intrinsic chemical properties to construct nanostructures, thereby improving physicochemical stability, enabling controlled release, and enhancing delivery efficiency and bioavailability at target sites [6,7]. Recently, the use of metal–phenolic networks (MPNs) has gained attention as a self-assembly strategy. Polyphenols such as TP, rich in catechol groups, can chelate various metal ions (e.g., Fe^3+^, Cu^2+^, Zn^2+^) to form stable three-dimensional network structures, allowing facile one-step synthesis of nanoparticles [8,9]. These MPNs not only provide a physical barrier that protects TP from environmental degradation but also often exhibit synergistic effects—where the incorporated metal ions (or their released forms, such as reactive oxygen species) enhance TP’s inherent antibacterial and antioxidant activities [10].

Among commonly used metal ions, Fe^3+^ stands out as an ideal candidate for constructing TP-based MPNs. It exhibits strong coordination affinity with TP, facilitating the formation of stable and uniform tea polyphenol–iron nanoparticles (TP-Fe NPs) [11,12]. Iron is also an essential trace element for humans, Fe^3+^ participates in redox reactions in biological systems and can catalyze the generation of reactive oxygen species (ROS), providing TP-Fe NPs with an additional antibacterial mechanism distinct from TP alone [10,13,14]. Studies have shown that, compared to free TP or MPNs formed with other metals (e.g., Cu^2+^), TP-Fe NPs often exhibit superior overall performance. First, they have a stronger ability to fight oxidation, which means they can effectively neutralize harmful free radicals and prevent fats from going rancid. Second, they are more powerful at killing or stopping the growth of a wide range of bacteria, including both common types known as Gram-negative and Gram-positive bacteria [15,16,17]. These attributes make TP-Fe NPs promising candidates for novel antimicrobial and antioxidant materials. Nevertheless, the effect of the TP/Fe^3+^ ratio on the morphology, size distribution, structural stability, and bioactivity of the resulting TP-Fe NPs remains insufficiently explored and warrants systematic investigation.

Nevertheless, a critical knowledge gap remains in systematically understanding how the TP/Fe^3+^ ratio precisely governs the structure–property relationships of the resulting TP-Fe NPs. Most studies have focused primarily on verifying synthetic feasibility rather than optimizing functional performance through precise stoichiometric control of the TP/Fe^3+^ ratio. Specifically, the effects of the TP/Fe^3+^ ratio on the coordination chemistry and structural stability of the TP-FeNPs, as well as its subsequent modulation of their antioxidant and antibacterial activities, remain inadequately explored. Additionally, while one-step co-precipitation is a known MPNs synthesis method, previous approaches often use harsh reaction conditions (e.g., extreme pH, organic solvents) or fixed metal-to-polyphenol ratios, limiting their applicability in food-related scenarios that demand biocompatibility and tunable performance.

Therefore, this study aimed to systematically optimize the preparation of TP-Fe NPs via a one-step co-precipitation method—distinct from existing approaches by (1) tuning the TP/Fe^3+^ ratio across a wide range (1:1 to 1:3 for fixed TP; 1:1 to 1:3 for fixed Fe^3+^), and (2) conducting synthesis under mild aqueous conditions (room temperature, pH 7.4) without additional cross-linkers or organic solvents to ensure biocompatibility. We comprehensively characterized the physicochemical properties (e.g., morphology, size, structure, and stability) of TP-Fe NPs and evaluated their antioxidant and antibacterial activities. This work not only deepens the understanding of structure–activity relationships in metal–phenolic nanomaterials but also offer a foundation for designing the efficient and safe food-active materials derived from natural bioactive substances.

## 2. Materials and Methods

### 2.1. Materials

All chemical reagents were of analytical grade and were used without further purification. Tea polyphenol (TP), 2,2-azino-bis (3-ethylbenzothiazoline-6-sulfonic acid) (ABTS) and 2,2-Diphenyl-1-picrylhydrazyl (DPPH) were purchased from Aladdin Biochemical Technology Co., Ltd. (Shanghai, China). Ferric chloride hexahydrate (FeCl_3_·6H_2_O) and potassium persulfate (K_2_S_2_O_8_) were obtained from Sinopharm Chemical Reagent Co., Ltd. (Shanghai, China). The microbial strains, *Escherichia coli* (*E. coli*, *ATCC25922*) and *Staphylococcus aureus* (*S. aureus*, *ATCC25923*), were supplied by the Food Microbiology Laboratory, College of Food Science, Fujian Agriculture and Forestry University (Fuzhou, China). Ultrapure water was employed throughout all experimental procedures, and all other chemicals used were of analytical grade. The L929 murine fibroblast cell was obtained from Punosai Life Technology Co., Ltd. (Wuhan, China). The Cell Counting Kit-8 (CCK-8) was purchased from Invigentech Inc. (South San Francisco, CA, USA). Live/dead cell staining kit was purchased from Bebo Biotechnology Co., Ltd. (Shanghai, China).

### 2.2. Preparation of TP-Fe NPs

According to the methods reported [18], TP-Fe NPs were synthesized using a one-step coordination-driven method. In a typical procedure, TP and FeCl_3_·6H_2_O were sequentially dissolved in deionized water at specific mass ratios to obtain different formulations: TP1-Fe1 NPs (TP:Fe = 1:1), TP1-Fe2 NPs (TP:Fe = 1:2), TP1-Fe3 NPs (TP:Fe = 1:3), TP2-Fe1 NPs (TP:Fe = 2:1), and TP3-Fe1 NPs (TP:Fe = 3:1). The mixture was stirred at 600 rpm at 45 °C for 1 h, after which the pH was adjusted to 10.0 using 0.5 M NaOH solution. The reaction was allowed to proceed for 12 h to facilitate the complete formation of nanoparticles. The resulting dispersion was subsequently freeze-dried to acquire the solid TP-Fe NPs for further use.

### 2.3. Yield, Encapsulation Efficiency, and Loading Capacity

The yield was calculated by dividing the weight of the TP Fe NPs obtained at the end by the total weight of all the starting materials used, and then converting this to a percentage. The supernatant containing free TP was analyzed by a UV-2600 spectrophotometer (Shimadzu, Kyoto, Japan), and its absorbance was recorded at 765 nm. With reference to the international standard ISO 14502-1:2005/Cor 1:2006 [19], the concentration of free TP was determined using a pre-established standard curve (y = 0.0122x + 0.0026, R^2^ = 0.9992). The encapsulation efficiency was calculated by measuring the concentration of unbound TP in the supernatant after centrifugation and washing. It is important to note that this calculated encapsulation efficiency might represent a slight overestimation, as some free TP could be physically adsorbed onto the nanoparticle pellet surface during the separation process. The yield, encapsulation efficiency (EE) and loading capacity (LC) of TP-Fe NPs were calculated using Equations (1)–(3):

(1) $$\mathrm{Yield}\;(\%)\;=\frac{m_{O}}{m_{T}+m_{F}}\;\times \;100\%$$

(2) $$\mathrm{EE}\;(\%)=\frac{m_{T}-m_{t}}{m_{T}}\;\times \;100\%$$

(3) $$\mathrm{LC}\;(\%)=\frac{m_{T}-m_{t}}{m_{T}+m_{F}}\;\times \;100\%$$

where $m_{O}$, $m_{T}$, $m_{t}$, and $m_{F}$ represent the mass of the TP-Fe NPs obtained, the initial mass of TP, the mass of free TP, and the initial mass of FeCl_3_·6H_2_O, respectively.

### 2.4. Particle Size, Polydispersity Index (PDI), and Zeta Potential

These three characterizations of the TP-Fe NPs were characterized using a Litesizer 500 analyzer (Anton Paar, Graz, Austria), based on the method of Wang [20]. Briefly, the TP-Fe NPs were dispersed in water at a concentration of 0.001 mg/mL and subjected to ultrasonication for 10 min. The resulting dispersion was then filtered through a 0.45 μm membrane filter prior to analysis. All measurements were performed in triplicate at 25 °C.

### 2.5. Scanning Electron Microscopy (SEM) and Transmission Electron Microscopy (TEM)

Using two types of microscopes, SEM (ZEISS Sigma 300, Oberkochen, Germany) and TEM (FEI Tecnai G2 F20, Hillsboro, OR, USA) characterized the morphology and surface structure of the TP-Fe NPs.

### 2.6. Fourier Transform Infrared Spectroscopy (FTIR)

FTIR spectroscopy of the TP-Fe NPs was performed on a Bruker Vertex 70 spectrometer (Karlsruhe, Germany) employing the KBr pellet technique. Spectra were collected from 4000 to 400 cm^−1^ at a resolution of 4 cm^−1^ with 32 accumulated scans.

### 2.7. X-Ray Diffraction (XRD)

The XRD pattern of the TP-Fe NPs was performed using a Rigaku SmartLab SE diffractometer (Osaka, Japan). The instrument scanned the TP-Fe NPs through an angle range from 10 to 80 degrees. This scan was done at a fixed speed of 5 degrees per minute.

### 2.8. Thermal Stability

The thermal stability of the TP-Fe NPs was evaluated under a nitrogen atmosphere using a thermogravimetric analyzer (Netzsch TG 209 F3, Selb, Freistaat Bayern, Germany). Heated the TP-Fe NPs from 30 to 800 °C at a steady pace of 10 °C per minute.

### 2.9. Water Contact Angle (WCA)

The hydrophilicity of the TP-Fe NPs was assessed by WCA measurement. Briefly, TP-Fe NPs (100 mg) were compressed using a tabletop hydraulic press (PC-12, Jingtuo, Tianjing, China) at a pressure of 10 MPa for 30 s. The static contact angle was then recorded using a JY-82C video contact angle instrument (Dingsheng, Chengde, China).

### 2.10. Color

The surface color of the TP-Fe NPs was measured using a CR-400 colorimeter (Konica Minolta, Kyoto, Japan), with the resultant data used to calculate the total color difference (ΔE) according to the following equation:

(4) $$\Delta E\;=\sqrt{{\left(L^{*}-L_{0}^{*}\right)}^{2}+{\left(a^{*}-a_{0}^{*}\right)}^{2}+{\left(b^{*}-b_{0}^{*}\right)}^{2}}$$

where $L^{*},\;a^{*},\;b^{*}$ are the color value of the TP-Fe NPs; $L_{0}^{*}{,\;a}_{0}^{*},\;b_{0}^{*}$ are the color value of the white paper.

### 2.11. Antioxidant Activity

The DPPH radical scavenging activity of the TP-Fe NPs was determined according to a method adapted from Zhao [21]. Briefly, 0.02 g of the TP-Fe NPs was ultrasonicated in 10 mL of water for 30 min to prepare an aqueous extract. Then, 1 mL of the extract was mixed with 9 mL of an ethanolic DPPH solution and allowed to react in the dark for 30 min. The absorbance of the resulting mixture was measured at 517 nm.

The ABTS radical scavenging activity of TP-Fe NPs was determined with slight modifications based on previous studies [22]. Briefly, the ABTS radical cation (ABTS^+^∙) solution was obtained by mixing ABTS (7 mM) solution and K_2_S_2_O_8_ (4.9 mM) solution, then stored in the dark at 25 °C for 16 h. The test extract was prepared by immersing 0.02 g of TP-Fe NPs in 10 mL of water and subjecting it to ultrasonic extraction for 30 min. For the assay, 1 mL of the extract was mixed with 9 mL of the diluted ABTS^+^∙ solution and allowed to react in the dark at for 15 min. The absorbance of the resulting mixture was immediately measured at 734 nm using a UV-2600 spectrophotometer (Shimadzu, Kyoto, Japan). The scavenging rates for both DPPH and ABTS radicals were calculated using the following equation:

(5) $$\mathrm{DPPH}\;\mathrm{radical}\;\mathrm{scavenging}\;\mathrm{rate}\;(\%)\;=\frac{A_{0}-A_{1}}{A_{0}}\times 100$$

(6) $$\mathrm{ABTS}\;\mathrm{radical}\;\mathrm{scavenging}\;\mathrm{rate}\;(\%)=\frac{B_{0}-B_{1}}{B_{0}}\times 100$$

where $A_{0}$ and $A_{1}$ represent the absorbance of the DPPH blank and sample, respectively; $B_{0}$ and $B_{1}$ represent the absorbance of the ABTS blank and sample, respectively.

### 2.12. Antibacterial Activity

The antibacterial activity of TP-Fe NPs against *E. coli* and *S. aureus* was evaluated using a modified colony counting method [23]. Briefly, 0.05 g of TP-Fe NPs was mixed with a bacterial suspension (approximately 4 × 10^6^ CFU/mL) and incubated at 37 °C for 2 h. Thereafter, 100 μL of the mixture was spread evenly on nutrient agar plates and cultured at 37 °C for 16 h [18]. The antibacterial rate was calculated based on the number of viable colonies compared to the control group. The bacterial survival rate was determined using Equation (7):

(7) $$\mathrm{Antibacterial}\;\mathrm{rate}\;(\%)\;=\frac{N_{0}-N_{1}}{N_{0}}\times 100$$

where $N_{1}$ represents the number of bacterial colonies in the TP-Fe NPs groups, and $N_{0}$ represents the number of bacterial colonies in the control group (without TP-Fe NPs).

### 2.13. In Vitro Cytotoxicity Assay

The cytotoxicity of the TP-Fe NPs on L929 mouse cells using a standard cell viability kit called CCK-8, according to an adapted method [24]. The TP-Fe NPs were sterilized under UV light for 30 min, ultrasonically dispersed in cell culture medium, and diluted to concentrations of 0.01 and 0.1 mg/mL. Prepared the cells by placing 7 × 10^3^ L929 cells into each well of a 96-well plate and letting them grow for a day. Then, exposed to 100 μL of nanoparticles suspensions for another 24 h, with TP-Fe NPs-free medium as the control. After incubation, cells were gently washed with PBS and incubated for 2 h with 100 μL of fresh medium containing 10% (*v*/*v*) CCK-8 reagent. The absorbance of the formazan product was measured at 450 nm using a microplate reader. All assays were conducted in triplicate.

To further confirm cell viability results, a complementary live/dead assay was performed. 1 × 10^5^ L929 cells per well were seeded in confocal dishes and cultured for one day. With TP-Fe NPs or control medium for one day, cells were washed with PBS and co-stained in the dark for 15 min at room temperature with a solution containing 2 μM Calcein-AM and 4.5 μM PI. Following three washes with PBS to remove excess dye, the cells were imaged using a fluorescence microscope at 100× magnification.

### 2.14. Statistical Analysis

All statistical analyses were performed using SPSS 26 statistical software. All experiments were conducted in triplicate (n = 3), and the data are expressed as mean ± standard deviation. Before the analysis, the homogeneity of variance test is used to ensure the accuracy of the analysis results. Differences between groups were analyzed by one-way ANOVA. When significant effects were found, Tukey’s Honest Significant Difference (HSD) post hoc test was used for multiple comparisons. *p* < 0.05 was considered statistically significant.

## 3. Results and Discussion

### 3.1. Analysis of Yield, Encapsulation Efficiency and Loading Capacity

**Figure 1:**
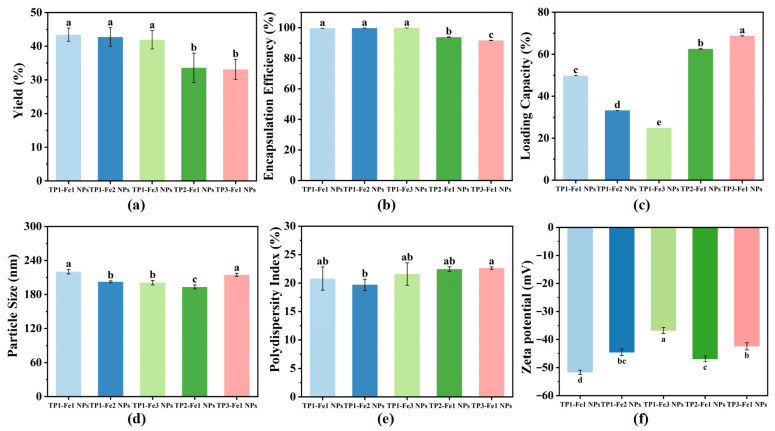
Characterization of the TP-Fe NPs: (**a**) yield; (**b**) encapsulation efficiency; (**c**) loading capacity; (**d**) particle size; (**e**) PDI; (**f**) zeta potential. Different lowercase letters represent significant differences among the TP-Fe NPs (*p* < 0.05).

Under conditions of constant TP content, the synthesis yield of TP-Fe NPs decreased from 43.41% to 41.90% with increasing iron content (Figure 1a). This reduction can be attributed to an excess of Fe^3+^, which promoted the formation of byproducts or aggregates rather than well-defined nanoparticles, thereby reducing effective reactant incorporation. Conversely, at fixed iron content, yield declined more substantially from 43.41% to 33.11% with increasing TP content. This trend implies that excessive TP may have intensified intermolecular interactions among polyphenols, compromising their dispersion and coordination with ratio [11]. Additionally, excess TP could have introduced competing reactions or steric hindrance, further suppressing nanoparticle formation and overall yield.

The calculated encapsulation efficiency of TP-Fe NPs was high across all formulations, which may be attributed to the potential physical adsorption of free TP onto the nanoparticle pellets during centrifugation. Despite the high values, the observed trends provided insights into the system’s behavior. The calculated encapsulation efficiency of TP-Fe NPs improved slightly from 99.65% to 99.81% with increasing iron content under conditions of constant TP content (Figure 1b). This enhancement likely stems from the reinforced cross-linking density and improved structural stability of the metal–phenolic network, which better retained TP within the nanoparticles and minimized leakage [25]. In contrast, at a fixed iron content, the calculated encapsulation efficiency exhibited a clear decrease from 99.81% to 91.71% as the TP content rose. This trend suggests that the encapsulation capacity of the TP-Fe NPs became saturated at higher TP ratios, leading to less efficient polyphenol incorporation.

A notable decline in loading capacity was observed when iron content increased under conditions of constant TP content, with values dropping from 49.82% to 24.95% (Figure 1c). This reduction can be explained by the greater mass contribution from non-active iron components, which diluted the relative proportion of TP in the nanoparticles [26]. On the other hand, at fixed iron content, loading capacity increased markedly from 24.95% to 68.78% with the increase in TP ratio, indicating that more TP molecules were successfully encapsulated without a proportional increase in carrier mass, thereby elevating the mass fraction of TP within the TP-Fe NPs.

### 3.2. Analysis of Particle Size, PDI, and Zeta Potential

The particle size and PDI of TP-Fe NPs remained consistent across different TP/Fe^3+^ ratios (Figure 1d,e), indicating that stoichiometric variations had minimal impact on these physical characteristics. In contrast, the Zeta potential was significantly influenced by the composition ratio (Figure 1f). TP1-Fe1 NPs demonstrated the highest absolute Zeta potential value (−51.70 mV), reflecting superior colloidal stability, while TP1-Fe3 NPs showed the lowest value (−36.82 mV), suggesting reduced stability and a greater propensity for aggregation [27]. This trend in Zeta potential can be explained by two main factors. First, under fixed TP content, increasing iron concentration likely neutralized the negatively charged phenolic groups on the nanoparticle surface, thereby reducing the absolute Zeta potential and potentially promoting particle aggregation. Second, at constant iron content, variations in TP content led to differences in molecular structure and phenolic hydroxyl density, which influenced both the coordination behavior with Fe^3+^ and the compactness of the surface coating layer. These structural differences ultimately modulated the surface charge density of the nanoparticles. In summary, the TP1-Fe1 ratio was identified as the optimal formulation for achieving stable TP-Fe NPs.

### 3.3. Surface Morphology Analysis

**Figure 2:**
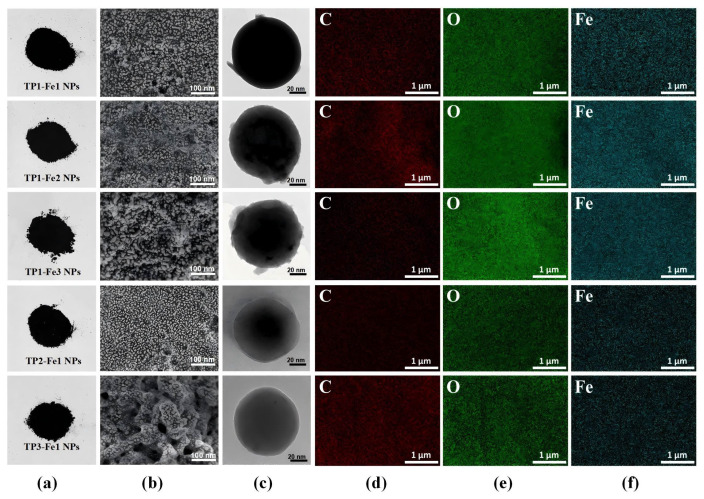
Structural and morphological characterization of TP-Fe NPs: (**a**) appearance, (**b**) SEM image, (**c**) TEM image; EDS elemental mapping images of TP-Fe NPs: C (**d**), O (**e**), and Fe (**f**).

A image depicting the appearance of the TP-Fe NPs is provided in Figure 2a. With increasing iron content, the TP-Fe NPs exhibited progressively evident aggregation behavior (Figure 2b). Initially TP1-Fe1 NPs gradually formed aggregates as the iron content rose from TP1-Fe1 NPs to TP1-Fe3 NPs, suggesting altered interparticle interactions. Similarly, an increase in TP content also induced aggregation in TP1-Fe1 NPs, TP2-Fe1 NPs, and TP3-Fe1 NPs. As natural antioxidants, TP may influence the dispersion state of nanoparticles through their interaction with particle surfaces, potentially modulating electrostatic repulsion or van der Waals forces between particles [18]. TEM images revealed that TP-Fe NPs maintained approximately spherical morphology without severe agglomeration in the observed fields (Figure 2c), indicating the synthesis methods successfully yielded well-dispersed nanoparticles. Elemental mapping and EDS analysis (Figure 2d–f, Table 1) further confirmed the successful synthesis of TP-Fe NPs, showing uniform distribution of C, O, and Fe. The weight proportion of iron increased from 14.15% to 30.95% with increasing iron content, whereas it decreased from 14.15% to 8.62% as the TP content rose.

### 3.4. FTIR Analysis

**Figure 3:**
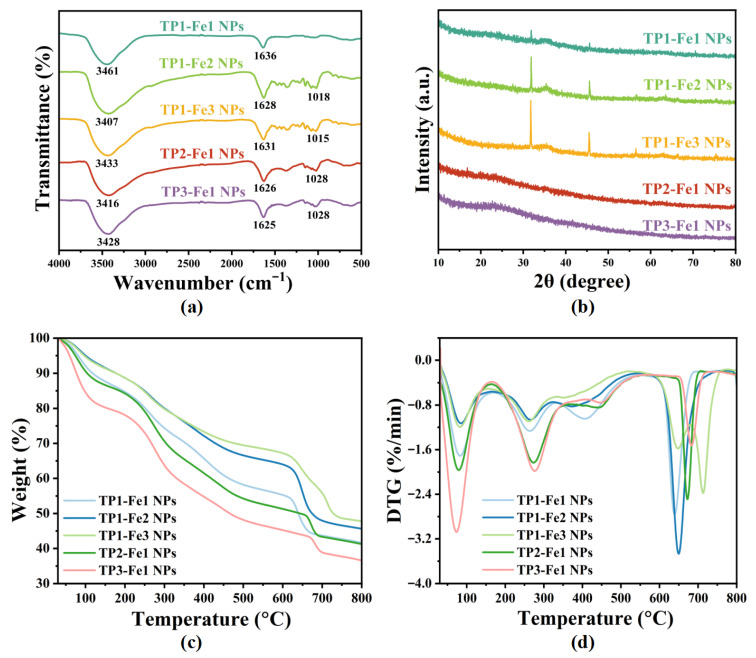
Structural and thermal characterization of TP-Fe NPs: (**a**) FT-IR spectra, (**b**) XRD patterns, (**c**) TG curves, and (**d**) DTG curves. The FT-IR spectra of the precursors (FeCl_3_·6H_2_O and TP) are provided in Figure S1. DTG (%/min) denotes the rate of mass loss (percentage of initial mass) per minute during thermal decomposition.

Figure 3a presents the FT-IR spectra of TP-Fe NPs. The corresponding spectra of the precursors, FeCl_3_·6H_2_O and TP, are provided in Figure S1 (Supporting Information) for comparison. As shown in Figure S1, the FeCl_3_·6H_2_O exhibits no characteristic absorption bands corresponding to C–O–C (1018–1028 cm^−1^) stretching vibrations—confirming that the band in the TP-Fe NPs spectra originate exclusively from TP. Furthermore, characteristic absorption bands attributable to Fe–O stretching vibrations—observed at 723 cm^−1^ (high-frequency region) and 457 cm^−1^ (low-frequency region) in the spectrum of FeCl_3_·6H_2_O [28], which is absent in the TP-Fe NPs spectra. This difference indicates that the coordination interaction between Fe^3+^ and TP functional groups modifies the local chemical environment of Fe^3+^. As shown in Figure 3a, all TP-Fe NPs exhibited characteristic absorption bands at 3407–3461 cm^−1^ (O–H stretching), 1625–1636 cm^−1^ (C=O stretching), and 1018–1028 cm^−1^ (C–O–C stretching) [29]. When the TP content was fixed, the O–H vibration to lower wavenumbers (from 3461 to 3407 cm^−1^) with increasing iron content, indicating enhanced coordination between Fe^3+^ and phenolic hydroxyl groups, which weakened the O–H bond strength. Concurrently, the C=O peak shifted from 1636 to 1625 cm^−1^, suggesting the involvement of carbonyl groups in coordination with Fe^3+^. When the iron content was fixed, the O–H band shifted to lower frequencies (e.g., from 3461 cm^−1^ in TP1-Fe1 NPs to 3416 cm^−1^ in TP3-Fe1 NPs) with increasing TP proportion, an effect attributed to intensified intermolecular hydrogen bonding arising from a greater number of free hydroxyl groups. Moreover, the intensity of the peak near 1028 cm^−1^ gradually increased with higher TP content, implying that a portion of phenolic hydroxyl groups remained uncoordinated under TP-rich conditions.

### 3.5. XRD Analysis

Broad diffraction features, indicative of an amorphous structure, were observed in the XRD patterns of five TP-Fe NPs samples (Figure 3b). Such loss of long-range order is a characteristic feature often associated with MPNs and provides initial structural evidence consistent with their formation. Notably, discernible diffraction peaks at approximately 30° and 46° were present in the pattern of the TP1-Fe1 NPs, TP1-Fe2 NPs and TP1-Fe3 sample, corresponding to crystalline elemental iron (Fe^0^). In contrast, these peaks were absent in the patterns of TP2-Fe1 NPs and TP3-Fe1 NPs. When the iron content was fixed, increasing the TP content progressively broadened the diffraction features, suggesting that TP not only suppressed the growth of iron-based crystallites but also fully inhibited detectable crystalline Fe^0^ formation at higher TP concentrations. This enhanced amorphization is closely related to the stronger coordination and encapsulation of iron species by the abundant phenolic hydroxyl groups in TP, a mechanism commonly observed in MPNs, as reported in related systems [30]. Conversely, under fixed TP content, higher iron content significantly increased the diffraction peak intensity, indicating greater aggregation of iron species within the amorphous matrix. However, the absence of distinct peak shifts implies no substantial change in the dominant iron valence state or local coordination environment. These structural features can be attributed to the one-step co-precipitation synthesis process: high TP content provides abundant coordination sites that encapsulate iron cores and disrupt long-range ordering, whereas high iron content promotes iron enrichment. In both cases, low reaction temperature and rapid precipitation kinetics collectively suppress crystalline framework development. Overall, the XRD data reveal an amorphous nanocomposite whose structural evolution with composition aligns well with the expected behavior of an in situ formed TP-Fe NPs.

### 3.6. TG Analysis

Thermogravimetric analysis (TGA) and derivative thermogravimetry (DTG) were employed to assess the thermal behavior of the TP-Fe NPs, thermogravimetric (TG) curve and DTG curve are shown in Figure 3c,d. The TP-Fe NPs samples exhibited typical multi-stage mass loss profiles over the temperature range of 30–800 °C. Under fixed TP content, the residual mass of the nanoparticles increased progressively with higher iron content, from 41.61% (TP1-Fe1 NPs) to 45.57% (TP1-Fe2 NPs) and 47.75% (TP1-Fe3 NPs), indicating that a higher iron content enhances the thermal stability of the material. In contrast, with the iron content fixed, the residual mass gradually decreased as the TP proportion increased—from 41.61% (TP1-Fe1) to 41.20% (TP2-Fe1 NPs) and further to 36.55% (TP3-Fe1 NPs., This phenomenon can be attributed to the relatively lower thermal stability of the organic TP component [31].

### 3.7. WCA Analysis

**Figure 4:**
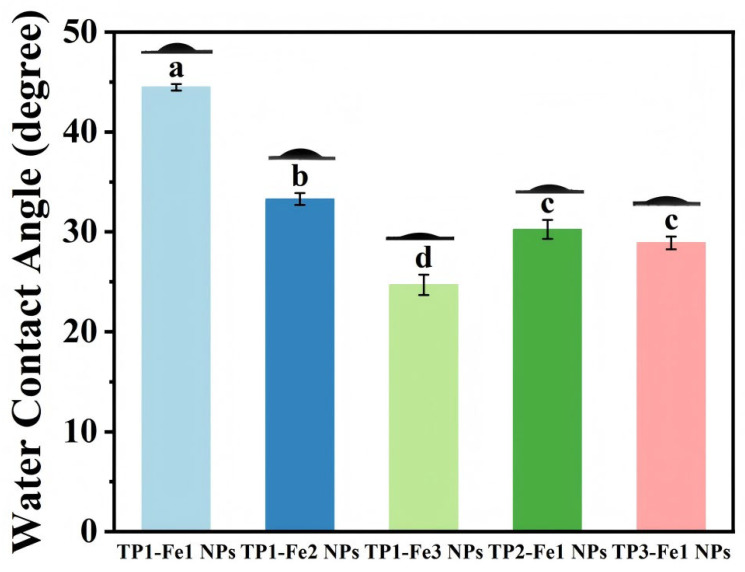
The water contact angle of TP1-Fe1 NPs, TP1-Fe2 NPs, TP1-Fe3 NPs, TP2-Fe1 NPs, TP3-Fe1 NPs. Different lowercase letters represent significant differences among the TP-Fe NPs (*p* < 0.05).

The influence of TP/Fe^3+^ ratios on surface wettability was evaluated by WCA measurements (Figure 4). With fixed TP content, increasing the iron concentration led to a systematic reduction in WCA, attributable to the elevated density of hydrophilic functional groups (e.g., hydroxyl and carbonyl) on the nanoparticle surface [32]. Likewise, with fixed iron content, a higher TP proportion also resulted in a lower WCA, likely due to the coordination with Fe^3+^ that enhanced surface hydrophilicity. These observations demonstrate that TP incorporation modulates the surface chemistry and microstructure of the nanoparticles, possibly by increasing the exposure of polar groups through coordination, thereby enhancing the material’s hydrophilicity.

### 3.8. Color Analysis

The color parameters of the TP-Fe NPs, detailed in Table 2, reveal distinct trends based on composition. Under fixed TP content, elevating the Fe^3+^ concentration led to a concomitant increase in L, a, and b* values, signifying a transition to lighter, more intensely reddish-yellow colors. Conversely, with fixed iron content, a higher TP content systematically decreased all color parameters. This visual darkening effect may be attributed to the increased surface roughness associated with higher TP content. This roughness alters the light-material interaction by facilitating diffuse scattering and broadening the absorption spectrum, thereby collectively reducing the perceived lightness, red intensity, and yellow saturation.

### 3.9. Antioxidant Activity Analysis

**Figure 5:**
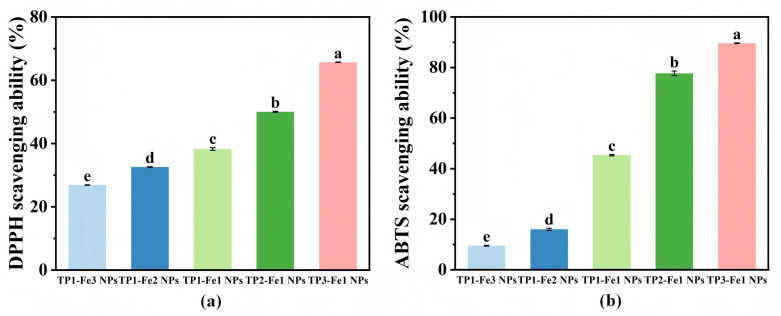
Antioxidant activity of TP-Fe NPs evaluated by (**a**) DPPH and (**b**) ABTS radical scavenging assays. Different lowercase letters represent significant differences among the TP-Fe NPs (*p* < 0.05).

The free radical scavenging capacity of TP-Fe NPs was evaluated at a fixed nanoparticle concentration via DPPH and ABTS assays, aiming to compare the relative effects of composition (TP/Fe ratio) on antioxidant activity (Figure 5). When the TP content was held constant, antioxidant activity decreased with increasing iron content. This can be attributed to the high iron content: an excessive amount of Fe^3+^ leads to the formation of a highly cross-linked and densely packed metal–phenolic networks. While this enhances the structural stability of the nanoparticles, it also results in the occupation of a majority of phenolic hydroxyl groups within the metal–phenolic network, thereby reducing the number of accessible active sites available for free radical scavenging, leading to a decline in antioxidant activity. Conversely, at a fixed iron content, TP-Fe NPs with higher TP proportions exhibited stronger antioxidant responses, which can be reasonably attributed to the greater abundance of phenolic hydroxyl groups in TP that serve as effective hydrogen donors [33]. As show in Figure 5, antioxidant activity consistently increased with higher TP content across the tested compositional series. The TP3-Fe1 NPs showed the highest scavenging rates, reaching 65.71% (DPPH) and 89.64% (ABTS), suggesting their potential as antioxidant candidates for food applications.

### 3.10. Antibacterial Activity Analysis

**Figure 6:**
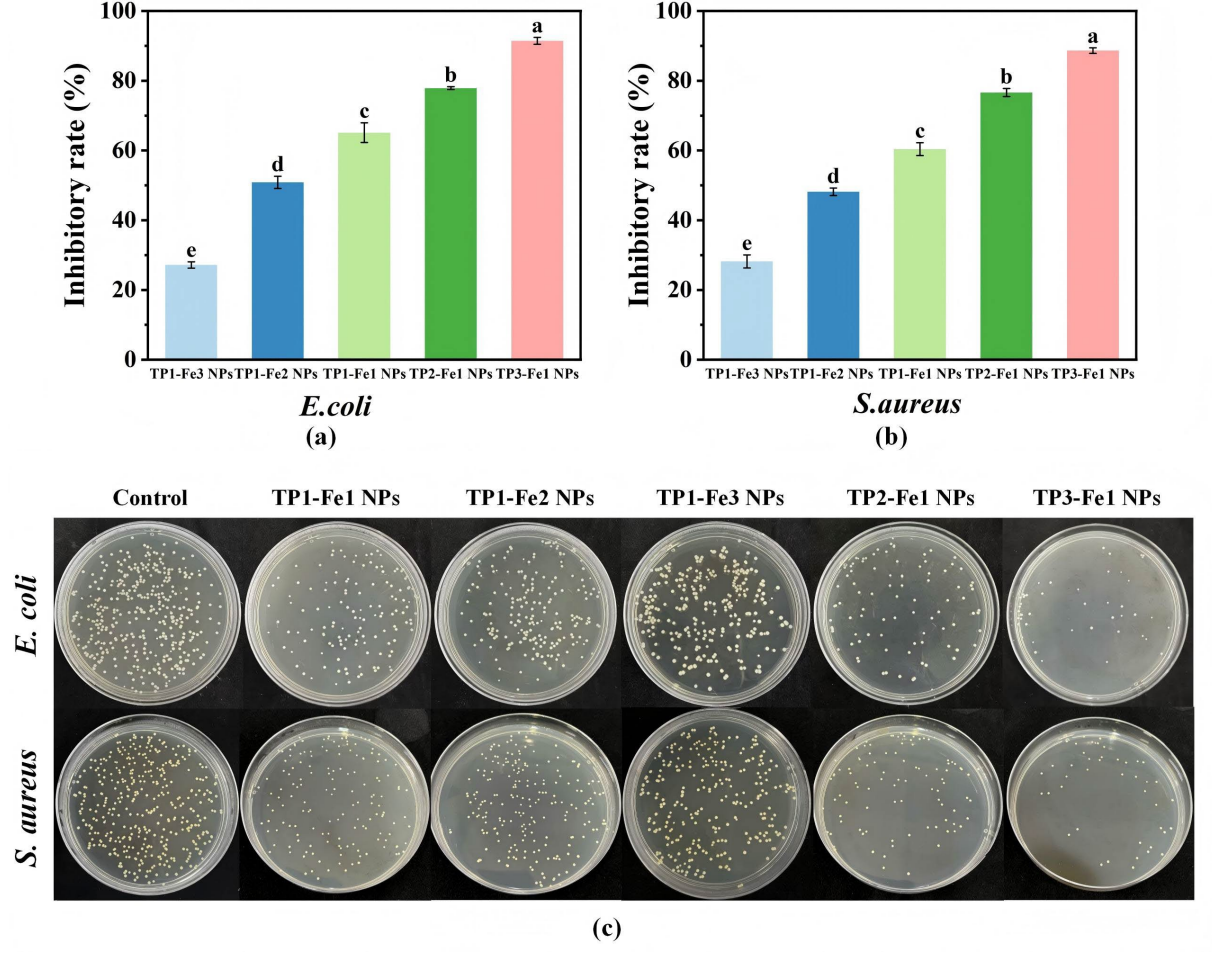
The inhibition rates of TP-Fe NPs on (**a**) *E.coli* and (**b**) *S.aureus*, and (**c**) the corresponding bacterial survival images. Different lowercase letters represent significant differences among the TP-Fe NPs (*p* < 0.05).

TP-Fe NPs exhibited composition-dependent antibacterial activity against both *E. coli* and *S. aureus* (Figure 6a,b). Under fixed TP content, increasing iron concentration resulted in reduced antibacterial efficacy, which may be explained by the compromised bioavailability of TP and the tendency of iron to aggregate or undergo redox changes at higher concentrations, thereby diminishing its synergistic antibacterial effect with TP. In contrast, when the iron content was constant, a higher TP proportion enhanced antibacterial performance. This enhancement is likely driven by the broad-spectrum antimicrobial activity conferred by TP. Based on established literature [34], the phenolic hydroxyl and catechol structures in TP are known to disrupt bacterial membrane integrity through interactions with lipids and proteins, a mechanism that is consistent with our observed results. Moreover, TP may interfere with essential metabolic pathways in bacteria, further suppressing their growth. These trends were corroborated by the bacterial survival results (Figure 6c). Therefore, the antibacterial action of TP-Fe NPs appears to involve a combination of the direct membrane-disrupting effect of TP and the potential supplementary effect of released iron species. Among all formulations, TP3-Fe1 NPs showed the strongest antibacterial activity, demonstrating their potential as efficient antimicrobial agents for food-related applications.

### 3.11. In Vitro Cytotoxicity Analysis

**Figure 7:**
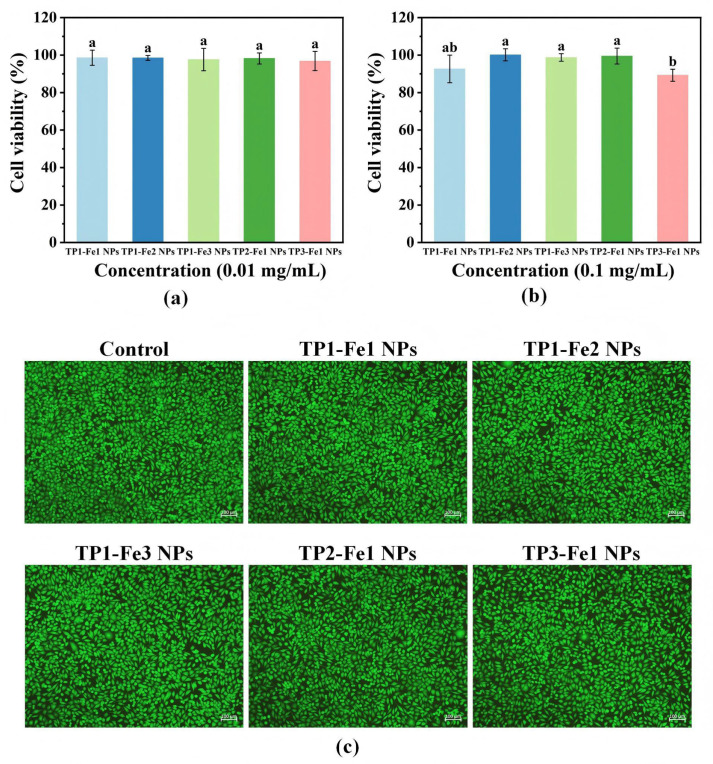
Cytotoxicity of the TP-Fe NPs at concentrations (**a**) 0.01 and (**b**) 0.1 mg/mL; the survival status of L929 cells when co-cultured with the sample for 24 h (100×) (**c**). Different lowercase letters represent significant differences among the TP-Fe NPs (*p* < 0.05).

Cytotoxicity of TP-Fe NPs was preliminarily assessed at two concentrations (0.01 and 0.1 mg/mL)—using L929 fibroblasts (a commonly utilized cell line for initial biocompatibility screening), as shown in Figure 7a,b. Cell viability remained consistently high (≥89%) across all TP-Fe NPs, with no significant cytotoxicity observed even for TP3-Fe1 NPs, which exhibited the strongest antibacterial activity. This finding indicates that the enhanced antibacterial performance was not achieved at the expense of cellular safety, providing preliminary evidence for the potential of these nanoparticles in food-related applications where biocompatibility is a critical prerequisite [35]. The morphological integrity and metabolic activity of L929 cells following 24 h of exposure to TP-Fe NPs were further confirmed by live/dead staining (Figure 7c). Cells in all treatment groups maintained normal spindle-shaped morphology, tight intercellular connections, and uniform distribution, closely resembling the control group. No apparent signs of apoptosis or necrosis—such as cell rounding, detachment, or membrane rupture—were detected. Additionally, the comparable fluorescence intensity between experimental and control groups also suggested well-preserved metabolic activity and cell membrane integrity.

## 4. Conclusions

This study successfully fabricated a series of TP-Fe NPs via coordination-driven self-assembly. Systematic investigation revealed that the TP/Fe^3+^ ratio significantly influenced their physicochemical properties. Comprehensive characterization confirmed near-spherical morphology with homogeneous elemental distribution and stable amorphous structure through Fe^3+^ coordination with phenolic hydroxyl/carbonyl groups. The optimal antioxidant and antibacterial activities observed in TP3-Fe1 NPs, which are primarily attributed to the phenolic hydroxyl groups of TP, resulted from their possession of the highest effective TP content. All TP-Fe NPs exhibited excellent biocompatibility with >89% cell viability in L929 cells and maintained cellular integrity. TP3-Fe1 NPs demonstrated the strongest bioactivity while preserving excellent biosafety, showing great potential as food-safe antibacterial materials for active packaging and preservation applications.

## Figures and Tables

**Table 1:** The elemental content of TP1-Fe1 NPs, TP1-Fe2 NPs, TP1-Fe3 NPs, TP2-Fe1 NPs, TP3-Fe1 NPs.

Nanoparticles	C Element (wt%)	O Element (wt%)	Fe Element (wt%)
TP1-Fe1 NPs	33.88	51.97	14.15
TP1-Fe2 NPs	33.56	47.36	19.08
TP1-Fe3 NPs	20.15	48.91	30.95
TP2-Fe1 NPs	48.23	41.26	10.51
TP3-Fe1 NPs	55.56	35.83	8.62

**Table 2:** Color parameters of TP1-Fe1 NPs, TP1-Fe2 NPs, TP1-Fe3 NPs, TP2-Fe1 NPs, TP3-Fe1 NPs.

Nanoparticles	L*	a*	b*	ΔE
TP1-Fe1 NPs	42.97 ± 0.17 ^c^	2.02 ± 0.10 ^c^	1.98 ± 0.17 ^c^	47.86 ± 0.17 ^b^
TP1-Fe2 NPs	43.43 ± 0.11 ^b^	2.22 ± 0.05 ^b^	2.44 ± 0.10 ^b^	47.43 ± 0.10 ^c^
TP1-Fe3 NPs	44.10 ± 0.09 ^a^	2.37 ± 0.05 ^a^	3.44 ± 0.16 ^a^	46.83 ± 0.10 ^d^
TP2-Fe1 NPs	42.11 ± 0.08 ^d^	1.11 ± 0.07 ^d^	1.14 ± 0.13 ^d^	48.67 ± 0.09 ^a^
TP3-Fe1 NPs	41.93 ± 0.04 ^e^	0.95 ± 0.03 ^e^	0.82 ± 0.05 ^e^	48.85 ± 0.04 ^a^

Different lowercase letters indicate significant differences among the TP-Fe NPs (*p* < 0.05).

## Data Availability

The original contributions presented in this study are included in the article/Supplementary Materials. Further inquiries can be directed to the corresponding authors.

## Supplementary Materials

The following supporting information can be downloaded at: https://www.mdpi.com/article/10.3390/foods14244337/s1, Figure S1: The FT-IR spectra of FeCl_3_·6H_2_O and TP.

## Abbreviations

TP	Tea polyphenols
TP-Fe NPs	tea polyphenol–iron nanoparticles
FeCl_3_·6H_2_O	Ferric chloride hexahydrate
K_2_S_2_O_8_	potassium persulfate
DPPH	2,2-Diphenyl-1-picrylhydrazyl
ABTS	2,2-azino-bis (3-ethylbenzothiazoline-6-sulfonic acid)
SEM	Scanning Electron Microscopy
TEM	Transmission Electron Microscopy
XRD	X-ray Diffraction
FTIR	Fourier Transform Infrared Spectroscopy
TG	Thermogravimetric
WCA	Water Contact Angle
CCK-8	Cell Counting Kit-8
PI	propidium iodide
