# Supplementary material for: One-Step Synthesis of Tea Polyphenol–Iron Nanoparticles for Enhanced Antioxidant and Antibacterial Properties
Source: Foods. 2025 Dec 16;14(24):4337. doi: 10.3390/foods14244337 (PMC12733163; doi:10.3390/foods14244337)
Supplement: Supplementary file 1 [file foods-14-04337-s001.zip › foods-4013975-supplementary.pdf]

Supplementary Materials

One-Step Synthesis of Tea Polyphenol-Iron Nanoparticles for Enhanced Antioxidant and Antibacterial Properties

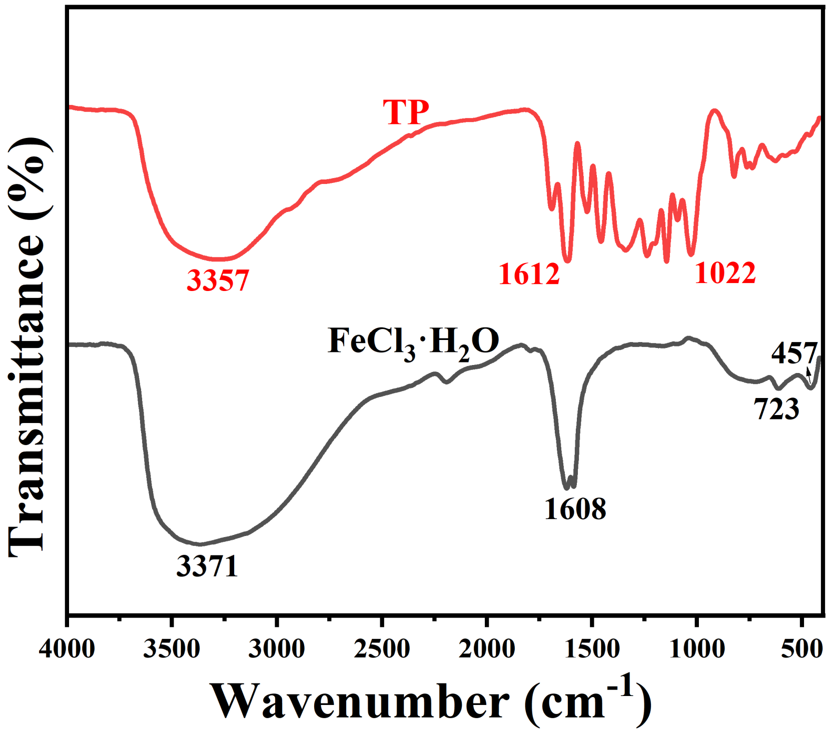

Figure S1. The FT-IR spectra of $FeCl_3{\cdot}6H_2O$ and TP.
